# Silica nanoparticles trigger the vascular endothelial dysfunction and prethrombotic state via miR-451 directly regulating the IL6R signaling pathway

**DOI:** 10.1186/s12989-019-0300-x

**Published:** 2019-04-11

**Authors:** Lin Feng, Xiaozhe Yang, Shuang Liang, Qing Xu, Mark R. Miller, Junchao Duan, Zhiwei Sun

**Affiliations:** 1 Department of Toxicology and Sanitary Chemistry, School of Public Health, Capital Medical University, Beijing, 100069 People’s Republic of China; 2 Beijing Key Laboratory of Environmental Toxicology, Capital Medical University, Beijing, 100069 People’s Republic of China; 3 Core Facilities for Electrophysiology, Core Facility Center, Capital Medical University, Beijing, 100069 People’s Republic of China; 4 University/BHF Centre for Cardiovascular Science, Queens Medical Research Institute, The University of Edinburgh, Edinburgh, UK

**Keywords:** Silica nanoparticles, Vascular endothelial dysfunction, Blood prethrombotic state, miR-451, IL6R signaling pathway

## Abstract

**Background:**

Safety evaluation is a prerequisite for nanomaterials in a wide range of fields, including chemical industries, medicine or food sciences. Previously, we had demonstrated that SiNPs could trigger the thrombotic effects in vivo, but the underlying mechanisms remain unknown. This study was aimed to explore and verify the role of miR-451a on SiNPs-induced vascular endothelial dysfunction and pre-thrombotic state.

**Results:**

The color doppler ultrasound results showed that SiNPs had the inhibitory effects on aorta velocity and cardiac output. The histological and ultrastructural analysis manifested that SiNPs could induce the vascular endothelial damage. In addition, the expression level of MDA was elevated while the activity of SOD and GSH-Px were decreased in aortic arch triggered by SiNPs, accompanied with the release of iNOS and decline of eNOS in blood serum. The immunohistochemistry results showed that the positive staining of TF and PECAM-1 were elevated in a dose-dependent manner induced by SiNPs. The activation of coagulation function occurred via shortened TT, PT and APTT while the FIB was elevated markedly induced by SiNPs. Coagulant factors (TF, FXa and vWF) and PLT numbers were increased whereas the levels of anticoagulant factors (ATIII, TFPI and t-PA) were decreased. Microarray analysis showed that the down-regulated miR-451a could target the gene expression of *IL6R*, which further activated the JAK/STAT signaling pathway triggered by SiNPs. Dual-luciferase reporter gene assay confirmed the directly target relationship between miR-451a and IL6R. Additionally, the chemical mimics of miR-451a led to attenuate the expression of IL6R/STAT/TF signaling pathway in vitro and in vivo induced by SiNPs, while the inhibitor of miR-451a enhanced the activation of IL6R/STAT/TF signaling pathway.

**Conclusions:**

In summary, SiNPs could accelerate the vascular endothelial dysfunction and prethrombotic state via miR-451a negative regulating the IL6R/STAT/TF signaling pathway.

**Electronic supplementary material:**

The online version of this article (10.1186/s12989-019-0300-x) contains supplementary material, which is available to authorized users.

## Background

Silica nanoparticles (SiNPs) were widely used in nanomedicine fields, including diagnosis, drug delivery systems, gene therapy vector and bioimaging [1]. According to the World Health Organization (WHO 2017), SiNPs are currently ranked as the No.2 largest production of all manufactured nanomaterials in the global market, with a volume of nearly 1.5 million tonnes (t) per year [2]. In addition, SiNPs as food additives have an acceptable daily intake (ADI) of 1.8 mg/kg [3]. Thus, this large-scale application has implicated the potential release of SiNPs into the environmental system. Consequently, SiNPs are also become one of the widely studied nanoparticles in nanotechnology environmental health and safety (nanoEHS). With the evidence showed that intratracheally instillation of SiNPs could cross the alveolar-capillary barrier and impair vascular homeostasis, cause the systemic inflammation and toxicological outcomes [4], there is an urgent required to explore and address the potential adverse effects of SiNPs on cardiovascular system [5].

Thrombosis is a key feature of pathological basis in the progression of severe cardiovascular diseases which resulting in myocardial infarction, ischemic stroke, deep vein thrombosis and pulmonary embolism [6]. The Virchow Theory which is widely acceptable for thrombus formation emphasizes that the thrombosis is closely associated with vascular endothelial injury, hemodynamic change and hypercoagulation [7]. Epidemiologic evidences found that long-term exposure to particulate matter could impact the coagulation function in the steelworkers via increasing the expression of endogenous thrombin and thrombus regulatory proteins [8]. A 10 μg/m^3^ elevation in particulate matter was associated with a 70% increase in deep vein thrombosis risk [9]. Since 76% of particulate matter is nanoscale particles which has the larger surface area for biological reactions, nanoparticles would be expected to produce more strikingly prethrombotic state in vivo than many microscale particles [10]. However, currently, few studies have focused on the relationship between nanoparticles and thrombus. Some studies showed that SiNPs could cause the aggregation of blood platelets and reduce the expression of plasminogen activator inhibitors in mice [4]. Recently, our study found that intravenous microinjection of SiNPs could lead to a hypercoagulable state in zebrafish embryos [11]. However, the underlying molecular mechanisms of SiNPs on thrombus formation remain unclear.

MicroRNAs (miRNAs) as composed of 19-25 nt small non-coding RNAs, are considered to recognize and combine the 3′-non coding region (3′-UTR) of target mRNA molecule, and affect the expression of target genes via degrading the mRNA or inhibiting the translation of target genes [12]. Recent studies have shown that miRNAs play important roles in a range of cardiovascular conditions, including arrhythmia, myocardial hypertrophy, hypertension, vascular lesions, atherosclerosis and thrombosis [13, 14]. It was reported that the endothelial progenitor cell-derived exosomes loaded with miR-126 could promote the deep vein thrombosis in mice [15]. MiR-145 facilitates the recanalization of arterial thrombosis via JNK signal pathway in a mouse model of cerebral infarction [16]. And the up-regulation of miR-495, miR-26a, miR-223 and miR-483-3p played the inhibition roles on thrombosis [17–20]. An recent study revealed that the miR-451 was closely linked with nonalcoholic fatty liver disease (NAFLD) which resulted in the increasing risk of cardiovascular diseases, metabolic diseases and type 2 diabetes [21]. Epidemiological studies had also showed that the expression level of miR-451 was positively correlated with coronary heart disease (CHD) [22]. Yet, to our best knowledge, there was no report on the relationship between miR-451 and coagulation function triggered by SiNPs.

Here, based on our previous study and high throughput screening of gene chip results, we explored the mechanism toxicity on the role of miR-451a in the process of thrombus formation induced by SiNPs. In addition, the IL6R/STAT/TF signaling pathways was further verified with the loss and gain function of miR-451a in vivo and in vitro. Our findings will provide new insight in the role of endothelial dysfunction and prethrombotic state triggered by nanoparticles, and may also provide critical molecular targets to screen the potential toxicity of nanoparticles.

## Methods

### Silica nanoparticles preparation and characterization

The Stöber technique was used to synthesize SiNPs, using 2.5 mL tetraethylorthosilicate (TEOS), 50 mL ethanol, 4 mL ammonia, and 2 mL water. The suspension was stirred at 200 rpm for 12 h at 40 °C, centrifuged at 12,000 rpm for 15 min at 4 °C, then washed three times with deionized water. SiNPs were dispersed in deionized water, and the size of 500 particles which selected randomly was measured using transmission electron microscopy (TEM) and Image J software. The zeta potential and hydrodynamic size of SiNPs were measured in distilled water, dulbecco’s modified eagle’s medium (DMEM), DMEM (with 10% serum) and normal saline by Zetasizer (Nano ZS90; Malvern, UK).

### Animal treatment

Six-week old male Sprague-Dawley (SD) rats were purchased from the Animal Experiments Center of Capital Medical University (Beijing, China) and maintained in a specific pathogen-free environment with free access to food and water, 24 ± 1 °C, 50 ± 5% RH and light/dark cycle per 12 h. Body weight range was 200 ± 20 g at the time of experiment. Experimental procedures were approved by the Animal Care and Use Committee in Capital Medical University with a permit number, AEEI-2016-087. Based on our previous study, rats were divided into four groups randomly: the control group (normal saline), and three concentrations of SiNPs (1.8 mg/kg·bw, 5.4 mg/kg·bw and 16.2 mg/kg·bw) [23]. Each group (*n* = 6) were received intratracheal instillation of suspension every three days for a 30 day period.

### Cells culture

Human umbilical vein endothelial cells (HUVECs) were cultured in Dulbecco’s modified eagle medium (DMEM) (Gibco, USA) with 10% fetal bovine serum (Gibco, USA) in 5% CO_2_ at 37 °C.

### Color Doppler ultrasonography

Doppler ultrasound using Vevo 2100 Imaging System (FUJIFILM VisualSonics Inc., USA) placed on the skin with sterile water-soluble gel at 45° inclination against its flow, and was used to measure descending aorta velocity and cardiac output (CO) at the 30th day. Flow velocity and electrocardiogram were recorded for a period of 60 s quiet free-breathing.

### Histopathological and ultrastructure examination

Aorta segments were isolated for pathological assesssment and fixed in 10% formalin solution for 48 h. The tissues were fixed into the 4% paraformaldehyde, embedded in paraffin, sectioned every 5 μm and stained with hematoxylin and eosin (H&E), orcein, and periodic acid Schiff (PAS) methods. Glutaraldehyde-fixed aortic slices were observed by a transmission electron microscope (TEM) (JEOL JEM2100, Tokyo, Japan) for assessment of tissue ultrastructure.

### Oxidative damage assessment

Peroxidation- and antioxidant-related indicators, such as malondialdehyde (MDA), superoxide dismutase (SOD) and glutathione peroxidase (GSH-Px), were measured in the rat aortic arch following the manufacturer’s protocols (Nanjing Jiancheng, China).

### NO/NOS system detection

Serum nitric oxide (NO) level was quantified by nitrate reduction according to the protocol from Beyotime Institute of Biotechnology in Shanghai, China. Blood was drawn from aortaventralis, then clotted for 2 h at room temperature and centrifuged at 2000 rpm at 4 °C for 20 min. The activation of endothelial nitric oxide synthase (eNOS) and inducible nitric oxide synthase (iNOS) in serum were detected by enzyme-linked immune sorbent assay (ELISA) kit according to the protocols (Cloud-Clone Corp. China). The absorbance was recorded by a microplate reader (Themo Multiscan MK3, USA) for NO at 540 nm and for eNOS and iNOS at 450 nm.

### Immunohistochemistry

4 μm-thick TMA slides were deparaffinized with antigen retrieval in 1 mM EDTA, pH 9.0 for 15 min. Tissue factor (TF) (Abcam, UK) and platelet endothelial cell adhesion molecule 1 (PECAM-1) (Abcam, UK) were applied at 1:100 in Sigel Stain antibody diluent (Cell Signaling Technology, MA, USA) for 1 h. Universal secondary antibody was applied for 15 min.

### Measurement of coagulation and fibrinolytic factors

Coagulation and fibrinolytic factors were evaluated in blood serum. Four indices of coagulation, including prothrombin time (PT), activated partial thromboplastin time (APTT), thrombin time (TT) and human fibrinogen (FIB), were to ascertain the endogenous/exogenous coagulation and plasmin function. All biochemical parameters were determined using commercial kits (Nanjing Jiancheng Bioeng Inst., China) according to the manufacturers’ instructions. Additionally, the serum levels of TF, coagulation factor X (FXa), vonwillebrand factor (vWF), antithrombin III (AT III), tissue factor pathway inhibitor (TFPI), tissue plasminogen activator (t-PA) and D-dimer (D2D) were determined using ELISA kits (Cloud-Clone Corp, Wuhan, China) under the absorbance of 450 nm.

### MiR-451a/IL6R detected by dual-luciferase reporter system

The 3′-UTR of corresponding gene containing miR-451a binding sites were amplified using overlap polymerase chain reaction (PCR) with prime sequence and cloned downstream of the dual-luciferase reporter gene assay in the pmirGLO vector (Promega, USA). 2.0 × 10^4^ HUVECs in 100 μL growth medium were maintained in 96-well plates, then were transfected with 100 ng reporter plasmids and 100 nmol/L miRNA mimics using Lipofectamine 3000 (Invitrogen, Carlsbad, CA, USA). The cells were harvested 48 h after transfection and were assayed using the dual-luciferase reporter gene assay kit (Promega, Madison, WI, USA) according to the manufacturer’s instructions. Dual-luciferase activities were normalized to firefly luciferase activities. Transfection was repeated in triplicate.

### Quantitative qRT-PCR assay

Bio-Rad software (CFX96™ optics module) was used to design optimal primer pairs for qRT-PCR. Total RNA was extracted from vessel issues using SV Total RNA Isolation System reagent (Promega, USA). The expression of miR-451a and the genes expression of *Jak1, Stat3, Tf, Il6r, Fib* and *Vwf* were performed by qRT-PCR kit (Takara, Japan) according to the manufacturer’s protocols. Raw data were normalized to the GAPDH values for each group, and the fold changes were shown as mean ± S.D. of six independent experiments. Primer used for qPCR were shown in Additional file 1: Table S1.

### Western blot assay

BCA protein kit (Pierce, USA) was used to extract the total cellular protein. Proteins were transferred to polyvinylidene fluoride membranes (Millipore, USA) by electrophoresis. The proteins of JAK1 (Abcam, UK), STAT3 (Abcam, UK), TF (Abcam, UK), IL6R (CST, USA), FIB (Abcam, UK) and vWF (Abcam, UK) were diluted 1000 times with TBS before use. Glyceraldehydes 3-phosphate dehydrogenase (GAPDH) was regarded as control. The cellulose membrane was blocked in Tris-buffered saline (with 5% non-fat milk powder) for 1 h at 25 °C, then incubated in the detected proteins overnight. After washed with 0.05% Tween-20 (TBST) three times, the cellulose membrane was incubated in horseradish peroxidase-conjugated anti-mouse/rabbit IgG secondary antibody (CST, USA) for 1 h at 25 °C, and washed with TBST three times. The relative densitometric analysis of antibody-bound proteins were calculated by Image J software.

### Loss and gain function of miR-451a measurement in vitro and in vivo

#### In vitro

MiR-451a mimics or inhibitors synthesized by Oligobio (Beijing, China) were used to upregulate or downregulate the expression level of miR-451a in HUVECs cells. Cells were seeded in six-well plates and were cultured to a density of 60%. Based on our previous results, 50 μg/mL concentration of SiNPs was significantly in cell toxicity via increasing concentration exposure in HUVECs cells [24]. The groups were as follow: control, mimics NC, inhibitor NC, SiNPs, miR-451a mimics + control, miR-451a mimics + SiNPs, miR-451a inhibitor + control and miR-451a inhibitor + SiNPs. Treatments were added into the plates with Lipofectamine 3000 (Invitrogen, Thermo Fisher, USA). After 24 h incubation, the transfection efficiency of miR-451a expression was confirmed using qPCR. After over- or low-expression cells of miR-451a were cultured in six-well plates for 24 h, then incubated with pmirGLO vector (Oligobio, Beijing, China) using Lipofectamine 3000. After incubation in normal culture atmosphere for 48 h, the expression levels of miR-451a and the genes expression of *IL6R, JAK1, STAT3, TF, FGA, FGB* and *FGG* were measured by qPCR assessment. The proteins of JAK1, STAT3, TF, IL6R and FIB were detected by western blot assay.

#### In vivo

MiR-451a mimics synthesized by Oligobio (Beijing, China) were used to upregulate miR-451a levels in male SD rats. The groups were as follow: negative control (NC), miR-451a mimics, SiNPs (16.2 mg/kg·bw), miR-451a mimics + SiNPs (16.2 mg/kg·bw). After given SiNPs through tracheal instillation for 5 times in SD rats, the expression levels of miR-451a and the genes expression of *Il6r, Jak1, Stat3, Tf and Vwf* were detected both in the blood serum and aortic arch. The protein levels of IL6R, JAK1, STAT3, TF and vWF were detected by western blot assay.

### Statistical analysis

One-way analysis of variance (ANOVA) and least significant difference (LSD) were used to determine the variance and compared different treatment groups by SPSS 23.0. *p < 0.05 was accepted as statistical significance.

## Results

### Characterization of SiNPs

**Fig. 1:**
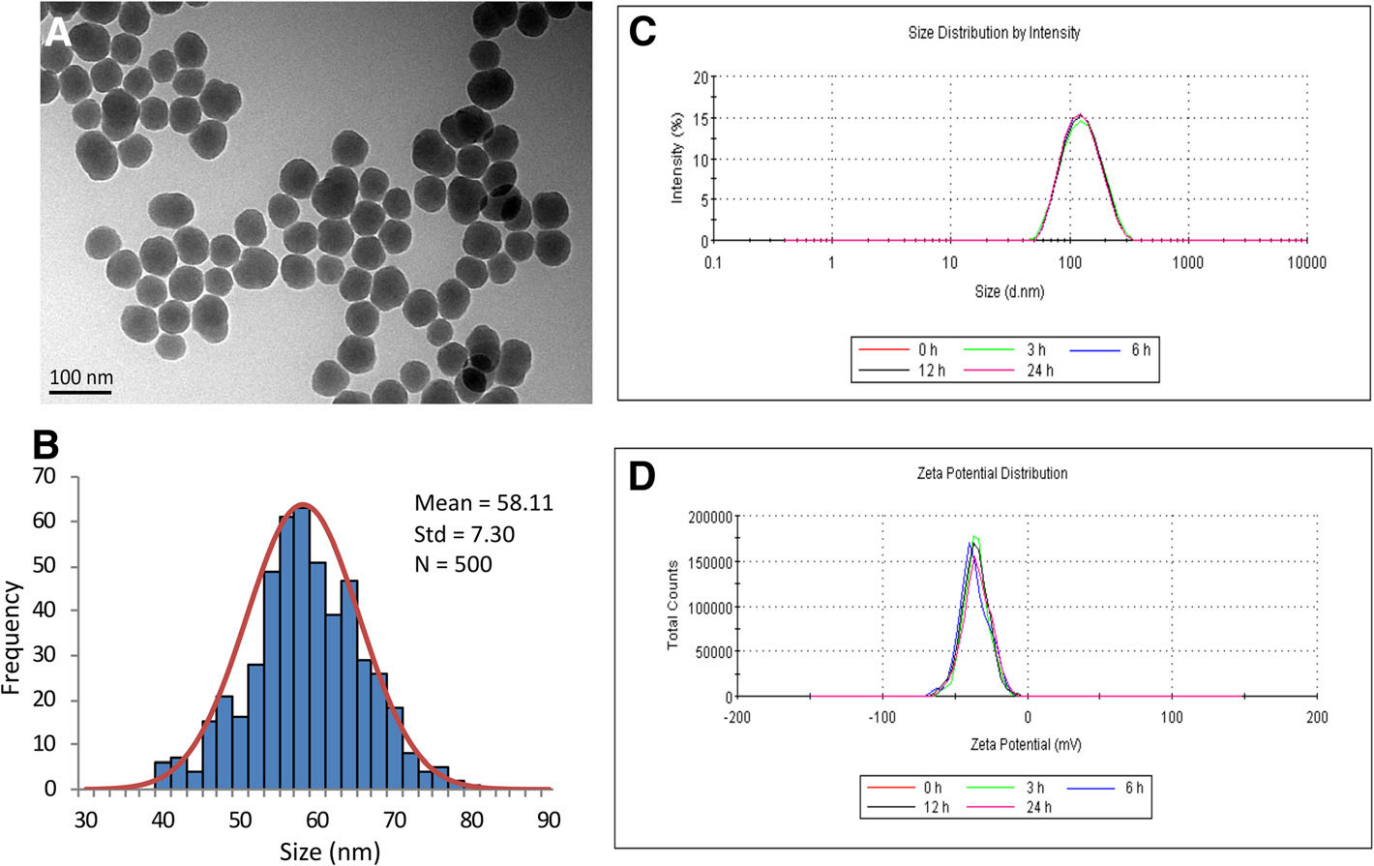
Characterization of SiNPs. (**a**) TEM images showed the spherical and well-dispersed particles. (**b**) Normal distribution diagram with a mean diameter of 58.11 ± 7.30 nm. (**c**) Size distribution by intensity. (**d**) Zeta potential distribution

SiNPs were near-spherical (Fig. 1a) with the average diameter of 58.11 ± 7.30 nm and a normal size distribution (Fig. 1b). Hydrodynamic size and zeta potential of SiNPs in different mediums are shown in Fig. 1c-d and Additional file 1: Table S2. Zeta potential was − 32.7 ± 1.7 mV in distilled water and − 30.8 ± 1.2 mV in normal saline. Our data demonstrated that SiNPs have good dispersibility in testing medium.

### Hemodynamic changes induced by SiNPs

**Fig. 2:**
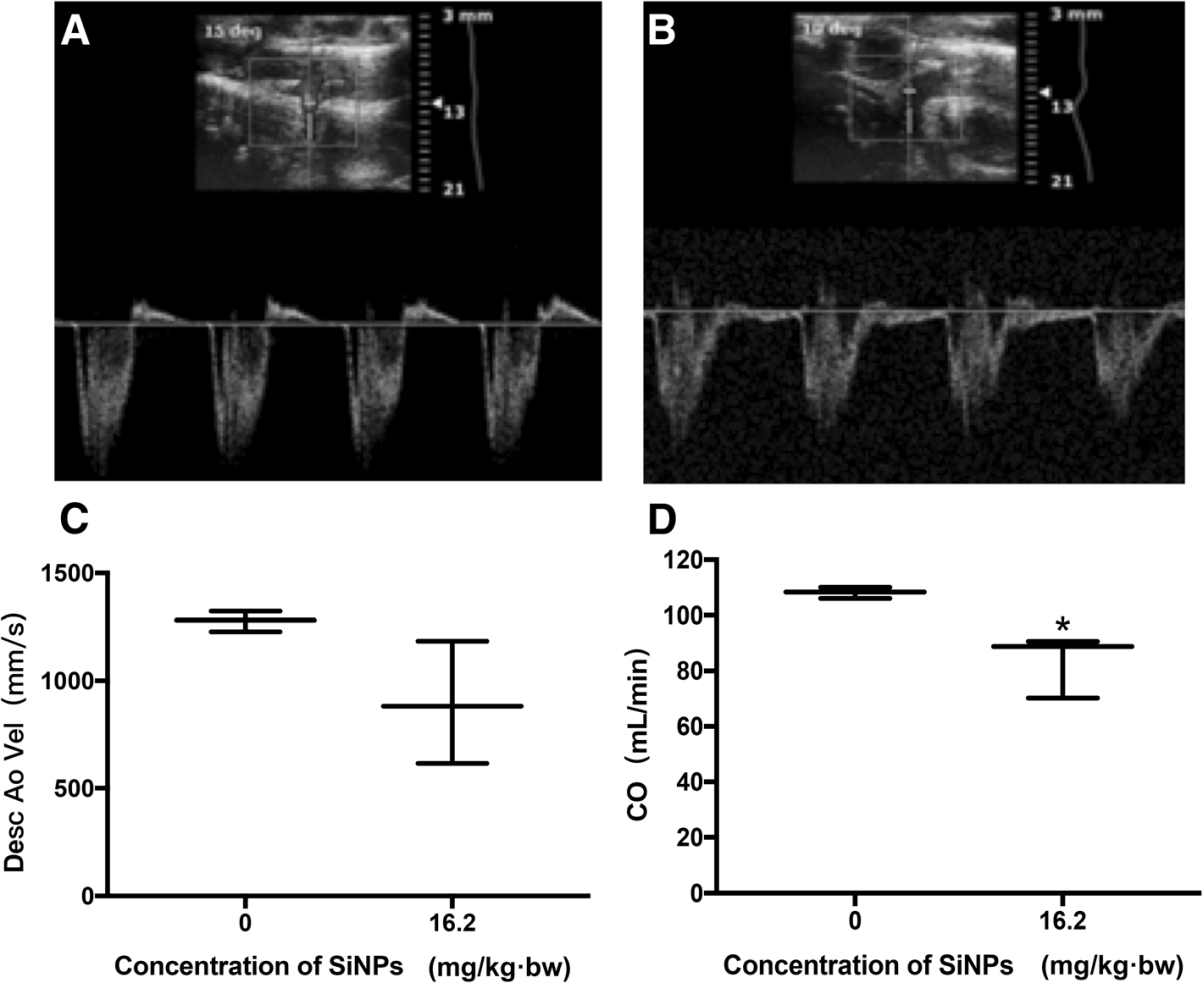
Hemodynamic changes induced by SiNPs. (**a**) Control (**b**) 16.2 mg/kg·bw (**c**) Descending aorta velocity. (**d**) Cardiac output. The descending aorta velocity and cardiac output were decreased from 1278 mm/s to 894 mm/s and from 108 mL/min to 83 mL/min compared with the control in 16 mg/kg·bw group induced by SiNPs. Data were expressed as means ± S.D. from six independent experiments. *p < 0.05 compared with control without SiNPs and any mimics/inhibitor

The blood flow velocity and blood flow volume were detected by Doppler ultrasound after exposure to SiNPs for 30 days (Fig. 2). Compared with control group, the descending aorta velocity was decreased from 1278 mm/s in control to 894 mm/s in SiNPs-treated group (16.2 mg/kg). In addition, the cardiac output was decreasing significantly from 108 mL/min to 83 mL/min in SiNPs-treated group compared with control. Our data from Doppler ultrasound indicated that SiNPs could decrease the blood flow velocity and change the hemodynamics.

### Histopathological and ultrastructural changes of vascular endothelium exposed to SiNPs

**Fig. 3:**
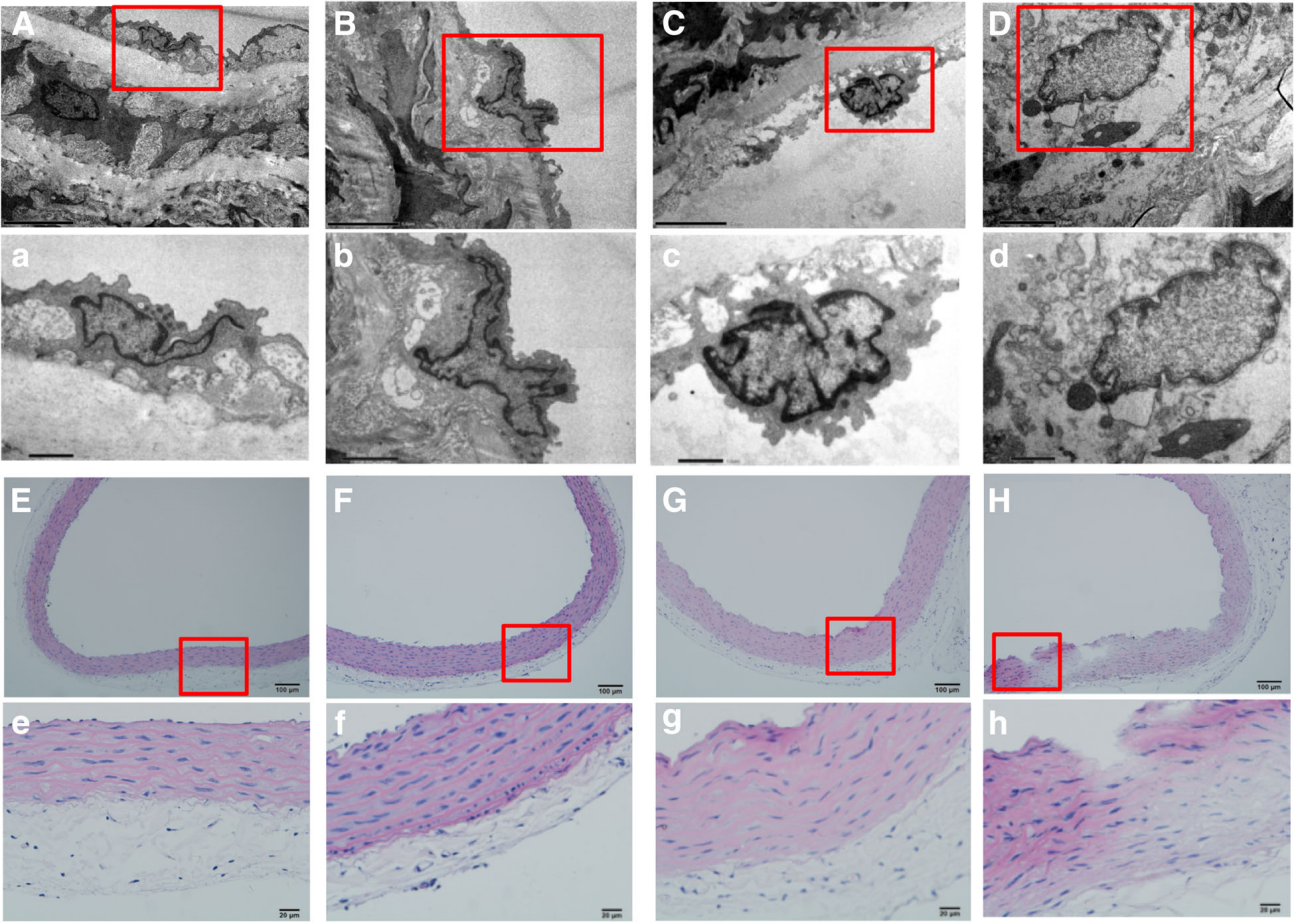
Histopathological and ultrastructural changes of vascular endothelium exposed to SiNPs. Results from the ultrastructure analysis showed that the ultrastructure of endothelial cells and basal membrane were integrated in control group (**a**) and 1.8 mg/kg·bw group (**b**). Local vacuolation of endothelial cells and the dissolution of nuclei were observed in 5.4 mg/kg·bw group (**c**). Swollen of mitochondria and the exfoliative endothelial cells were observed in 16.2 mg/kg·bw group (**d**). Morphology of aortic arch was normal in both control group (**e**) and 1.8 mg/kg·bw group (**f**), whereas inflammatory cells and edema appeared in 5.4 mg/kg·bw group (**g**) and 16.2 mg/kg·bw group (**h**)

Results from the ultrastructure analysis showed that the normal structure of vascular endothelium were observed in the aortic arch of the control group (Fig. 3a) and the low dose of SiNPs-treated group (Fig. 3b). While the vacuolation of endothelial cells and the dissolution of nucleus were observed in medium dose group (Fig. 3c). And in the high dose of SiNPs-treated group, the swollen mitochondria and exfoliative endothelial cells were occured (Fig. 3d). The histopathological assessment showed that although the morphology of aortic arch was normal in both control group (Fig. 3e) and low dose group (Fig. 3f), there was a number of inflammatory cells, aortic wall and tunica media thickness were occurred in the higher dose of SiNPs-treated groups (Fig. 3g and h). Taken togerther, the results illustrated that SiNPs could cause vascular endothelial injury in vivo.

### Effects of SiNPs on vascular oxidative damage, NO/NO system and vascular function

**Fig. 4:**
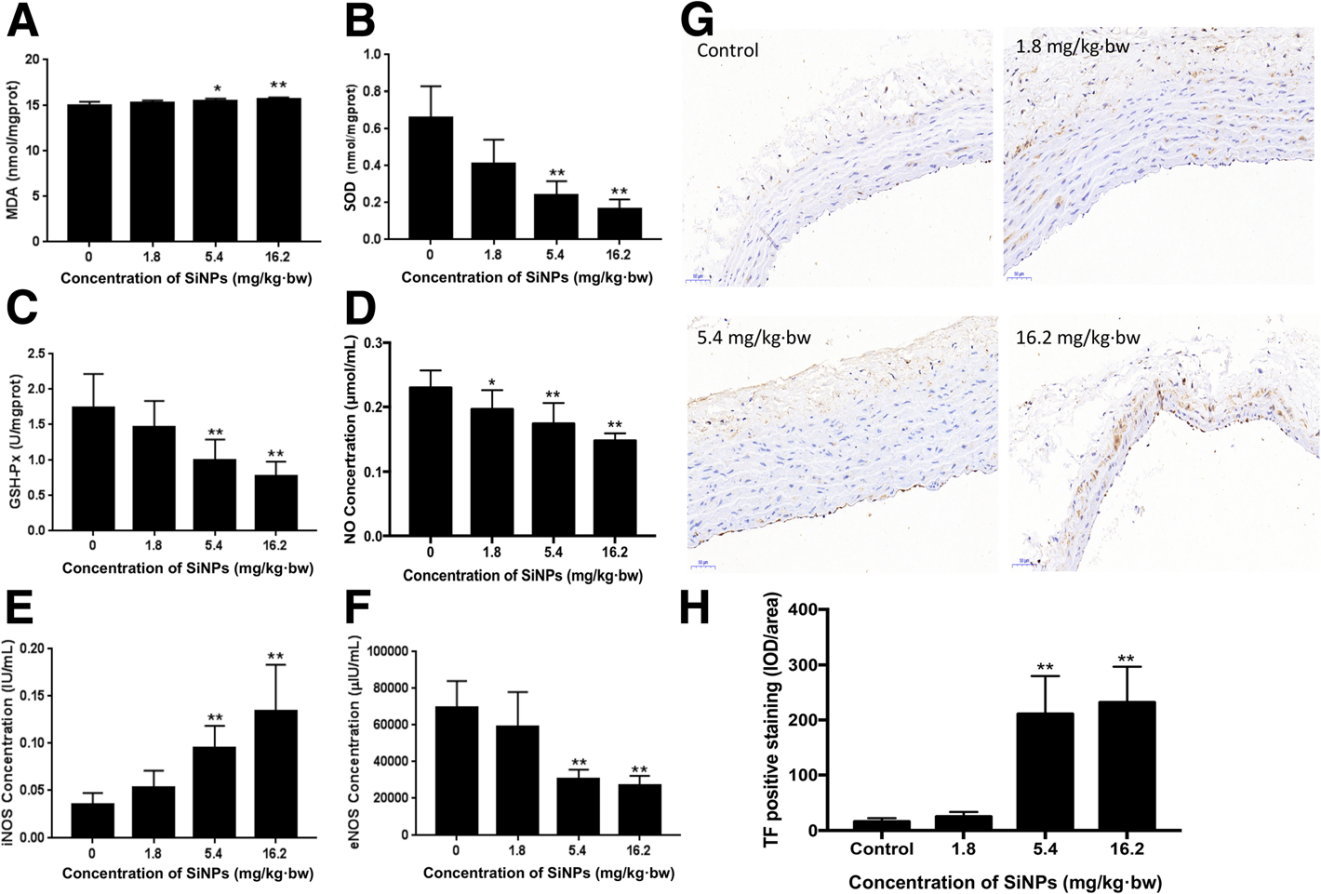
Effects of SiNPs on vascular oxidative damage, NO/NO system and vascular function. The level of MDA (**a**) was increased, while the activities of SOD (**b**) and GSH-Px (**c**) were decreased in a dose dependent manner. The activity of NO (**d**) and eNOS (**f**) decreased markedly and the activity of iNOS (**e**) increased with increasing SiNPs. Effect of SiNPs on tissue factor (TF) expression in aortic arch sections were detected by immunohistochemical analysis (**g**). Expression of TF increased in a dose-dependent manner by SiNPs (**h**). Data were expressed as means ± S.D. from six independent experiments. *p < 0.05 and **p < 0.01 compared with control without SiNPs and any mimics/inhibitor

The oxidative damage, NO/NOS system and tissue factor (TF) expression were measured in the aortic arch induced by SiNPs (Fig. 4). The lipid peroxidation levels of MDA were increased significantly compared with control group; whereas the antioxidant activities of SOD and GSH-Px were decreased markedly in a dose-dependent manner (Fig. 4a, b and c). In addition, the serum levels of NO and eNOS were decreased significantly, while the activity of iNOS was increased in a dose dependent manner after exposed to SiNPs(Fig. 4d, e and f). What’s more, the immunohistochemistry results showed that the positive cell staining of TF was mainly located in the vascular endothelium (Fig. 4g). Notably, as shown in Fig. 4h, the protein level of TF in high dose of SiNPs-treated group was significantly elevated (14.5-fold higher than that in control). And the number of PECAM-1 positive cells were also increased in vascular endothelium (Additional file 1: Figure S1). Taken together, our data demonstrated that SiNPs caused the vascular oxidative damage, disturbed the NO/NOS system and impaired the endothelial function.

### Effects of SiNPs on coagulation function

**Fig. 5:**
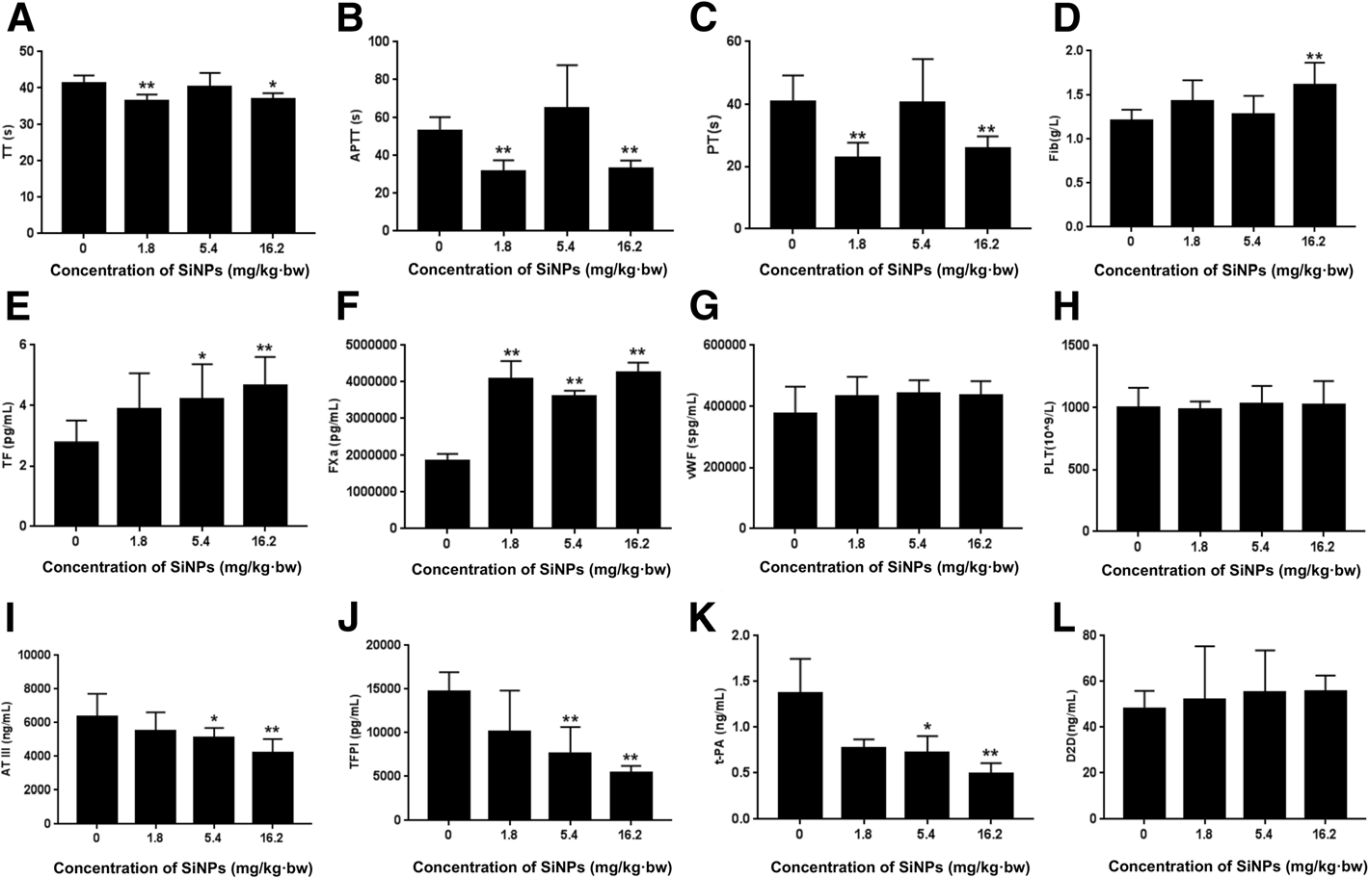
Effects of SiNPs on coagulation function. Decreased TT (**a**) and APTT (**b**) demonstrating a trend towards increased blood coagulation in response to high concentration of SiNPs. Increased FIB (**c**) and shortened PT (**d**) are associated with increased blood viscosity and hypercoagulation. Increased TF (**e**) and FXa (**f**) demonstrated the activation of exogenous and endogenous coagulation systems. Increased vWF (**g**), PLT (**h**) and D2D (**l**) were detected. The anti-coagulative and fibrinolytic system were inhibited by decreasing AT III (**i**), TFPI (**j**) and t-PA (**k**). Data were expressed as means ± S.D. from six independent experiments. *p < 0.05 and **p < 0.01 compared with control without SiNPs and any mimics/inhibitor

Coagulant factors (TF, FXa, D2D and vWF) and anticoagulant factors (ATIII, TFPI and t-PA) were measured in serum after SD rats exposed to SiNPs for 30 days (Fig. 5). Decreased TT and APTT were evidented in some concentrations of SiNPs. Decreased PT and elevated FIB were also observed after treatment with SiNPs. The expression of TF, FXa, vWF and D2D elevated, while the expression of AT III, TFPI and t-PA decreased in dose-dependent of SiNPs. Our results indicated that SiNPs could impact the coagulation function via activating the endogenous/exogenous coagulation system and fibrinolytic system.

### Verification of miR-451a/IL6R target regulatory relationship

**Fig. 6:**
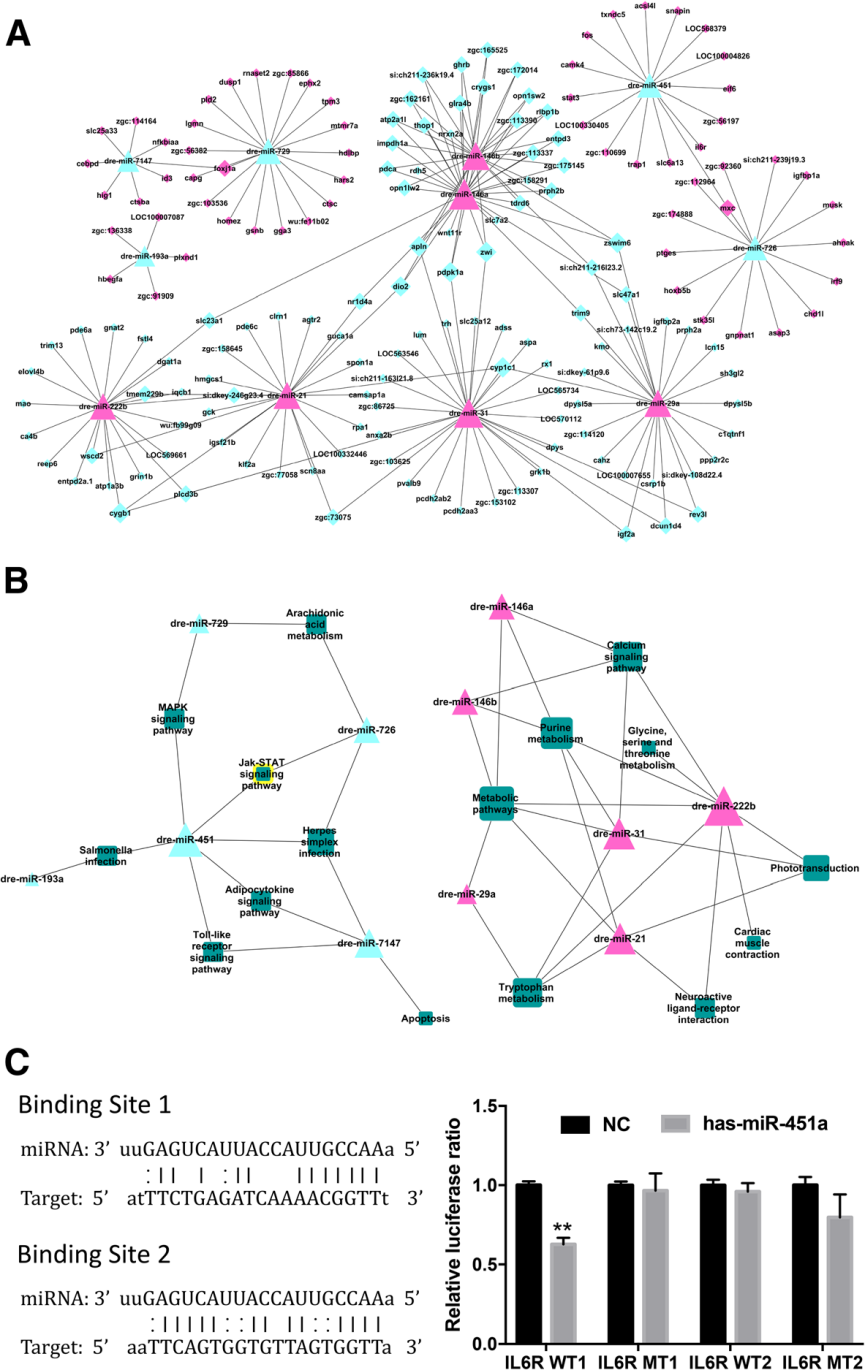
Microarray analysis and verification of miR-451a/IL6R induced by SiNPs. (**a**) MicroRNA-Gene-Network. (**b**) MicroRNA-Pathway-Network. (**c**) Dual-luciferase report gene assay verified the target regulatory relationship between miR-451a and *IL6R* through pmiR-IL6R-WT1 3′-UTR binding site

As shown in Fig. 6a and Additional file 1: Table S3, microarray and bioinformatics analysis revealed that miR-451a has the possible target regulatory relationships with several genes, such as il6r, stat3, acsl4l, fos, txndc5, and so on. miR-451 was associated with JAK-STAT signaling pathway, Adipocytokine signaling pathway, and Toll-like receptor signaling pathway (Fig. 6b and Additional file 1: Table S4). After transfected with miR-451a, the significant inhibition occurred in the expression of luciferase which contains *IL6R* site 1. On the contrary, after the mutation of target point, miR-451a could not inhibit the expression of luciferase, and miR-451a had no inhibit effects on the expression of luciferase which contains *IL6R* site 2 (Fig. 6c). Dual-luciferase reporter gene assay confirmed the directly target regulatory relationship between miR-451a and IL6R.

### The role of SiNPs-triggered miR-451a on IL6R/STAT/TF signaling pathway in vivo

**Fig. 7:**
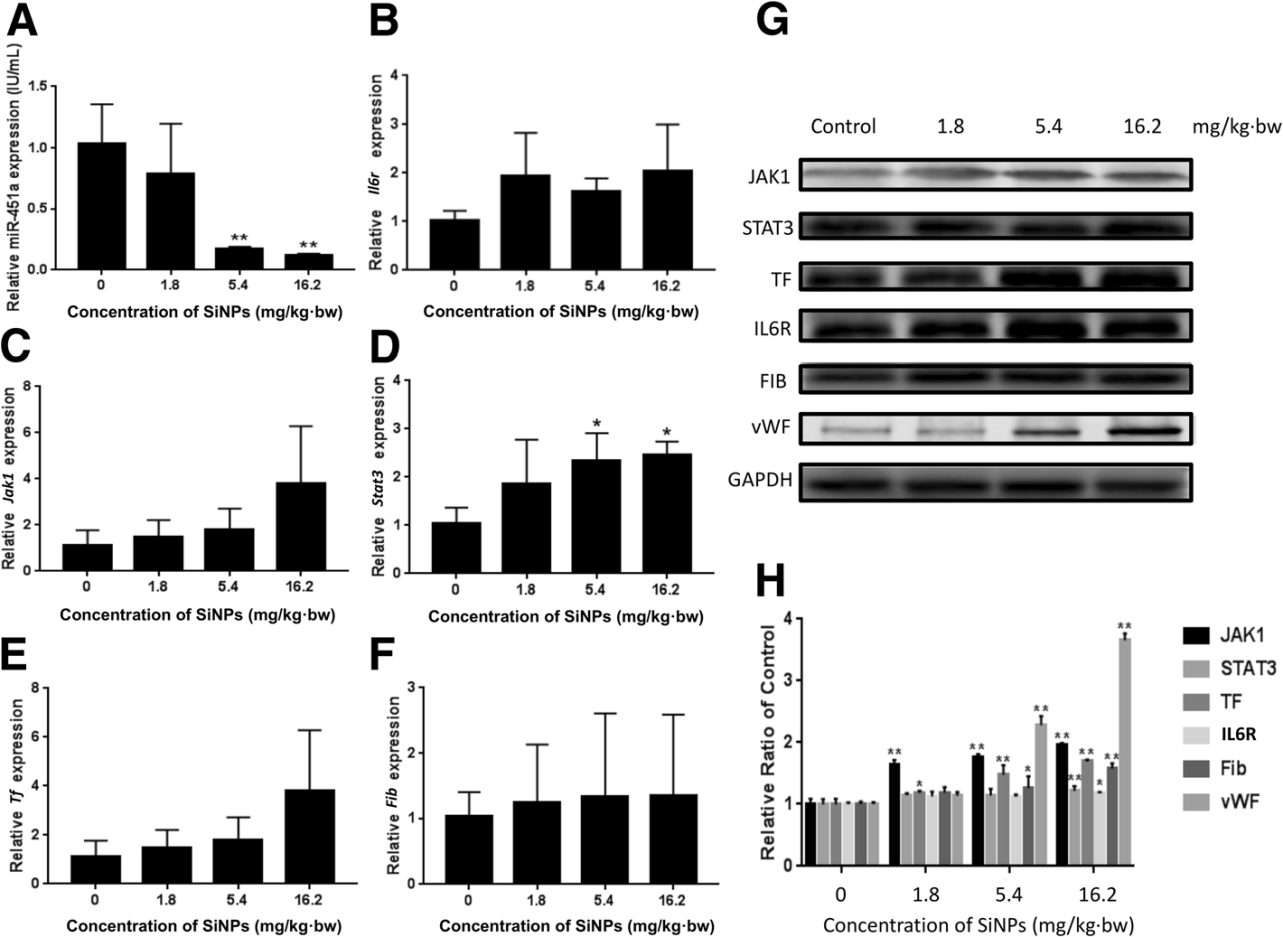
The role of SiNPs-triggered miR-451a on IL6R/STAT/TF signaling pathway in vivo. Decreased miR-451a and increased genes expression of *Il6r, Jak1*, *Stat3*, *Tf* and *Fib* were detected after SiNPs exposure (**a**-**f**). Protein levels of JAK1, STAT3, TF, IL6R, FIB and vWF were significantly increased in a dose-dependent manner induced by SiNPs (**g**). The protein levels of JAK1, STAT3, TF, IL6R, FIB and vWF were caculated by the relative densitometric analysis (**h**). Data are expressed as means ± S.D. from six independent experiments. *p < 0.05 and **p < 0.01 compared with control without SiNPs and any mimics/inhibitor

**Fig. 8:**
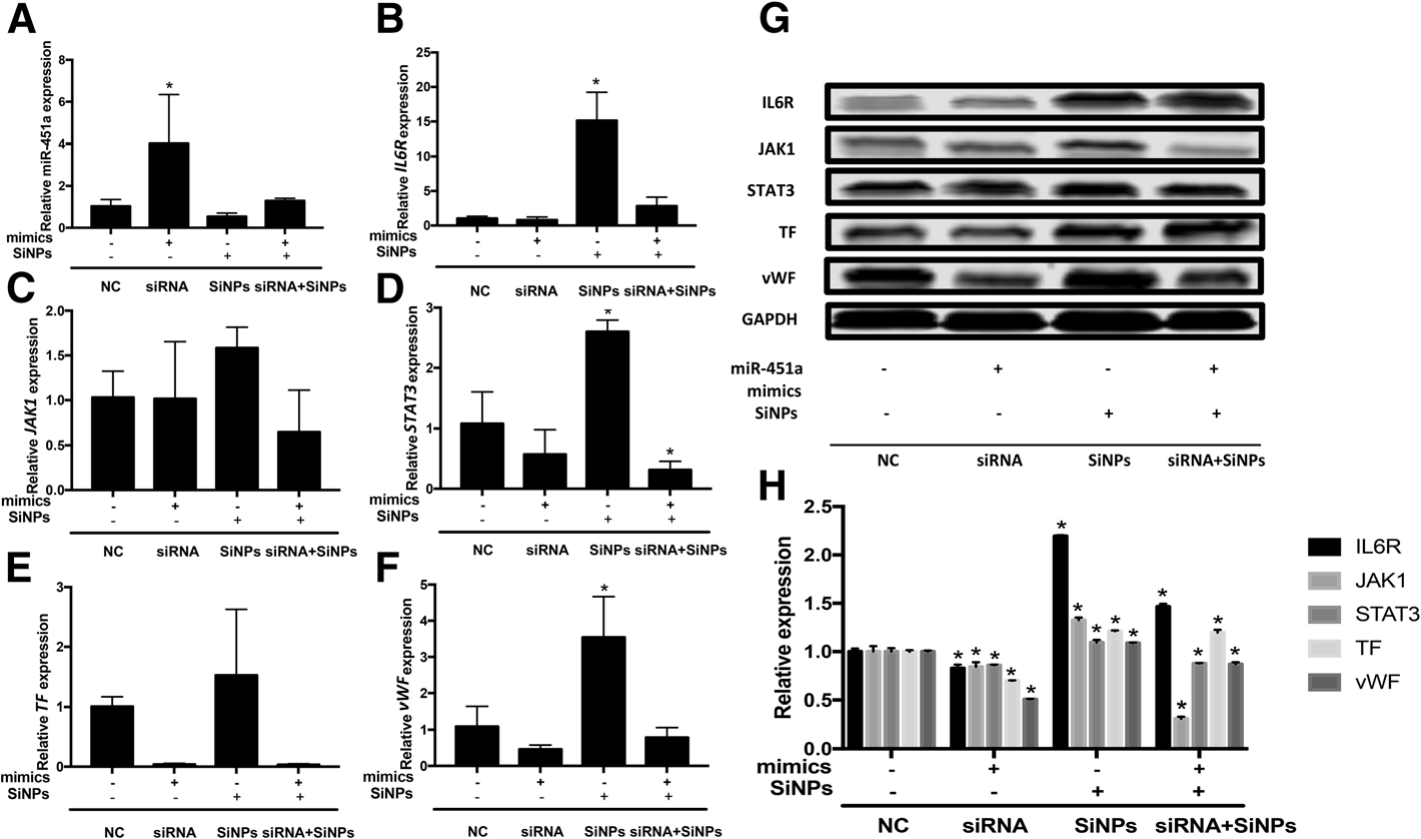
The effects of plasmid transfection with miR-451a mimics in SD rats exposed to SiNPs. Decreased miR-451a and increased genes expression of *IL6R, JAK1*, *STAT3*, *TF* and *vWF* were detected after SiNPs exposure (**a**-**f**). Protein levels of IL6R, JAK1, STAT3, TF and vWF were significantly decreased in miR-451a mimics + SiNPs group (**g**). The protein levels of IL6R, JAK1, STAT3, TF and vWF were calculated by the relative densitometric analysis (**h**). Data are expressed as means ± S.D. from six independent experiments. *p < 0.05 and **p < 0.01 compared with negative control group

On the one hand, SiNPs could downregulate the expression of miR-451a and upregulate the genes expression of *Jak1*, *Stat3*, *Tf*, *Il6r* and *Fib* in SD rats’ aortic arch (Fig. 7a-f). Similar trends were observed in the related protein expression induced by SiNPs (Fig. 7g-h). The high concentration of SiNPs showed more obvious changes in these indicators of endothelial injury. On the other hand, the results of transfection experiment in SD rats manifested that the increased expression of miR-451a were significantly in miR-451a mimics + control group and miR-451a mimics + SiNPs group compared with the control. The expression of gene *Il6r* increased in SiNPs groups but decreased in miR-451a mimics group. The genes expression of *Jak1*, *Stat3*, *Tf* and *Vwf* had the similar changes with *Il6r* which showed the activation of IL6R/JAK/STAT/TF signaling pathway (Fig. 8a-f). The proteins of IL6R, JAK1, STAT3, TF and vWF were detected which showed the consistent trends with qPCR analysis (Fig. 8g-h). The results in vivo manifested that SiNPs could decrease miR-451a and activate the IL6R signaling pathway.

### The role of SiNPs-triggered miR-451a on IL6R/STAT/TF signaling pathway in vitro

**Fig. 9:**
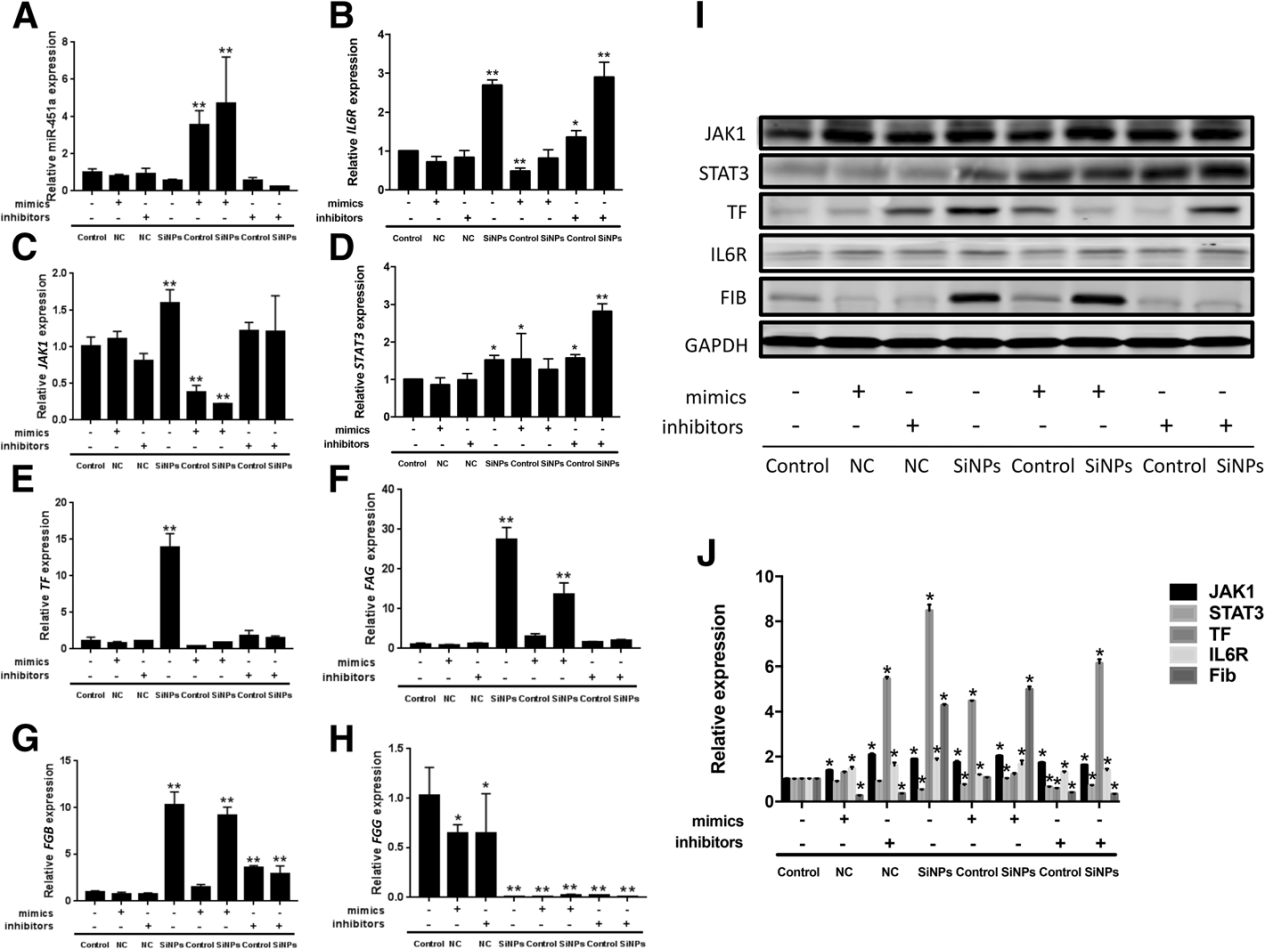
The role of SiNPs-triggered miR-451a on IL6R/STAT/TF signaling pathway in vitro by miR-451a mimics/inhibitor. The expressions of miR-451a, *IL6R*, *JAK1*, *STAT3*, *TF*, *FGA*, *FGB* and *FGG* (**a**-**h**) were measured in HUVECs. The results of plasmid transfection with miR-451a mimics/inhibitor on IL6R/STAT/TF pathway (**i**). The protein levels of IL6R, JAK1, STAT3, TF and FIB were caculated by the relative densitometric analysis (**j**). Data are expressed as means ± S.D. from three independent experiments. *p < 0.05 and **p < 0.01 compared with control without SiNPs and any mimics/inhibitor

As shown in Fig. 9, the expression of mimics/inhibitor NC were almost the same with the control group without SiNPs. The expression of miR-451a was decreasing in SiNPs group, miR-451a inhibitor + control group and miR-451a inhibitor + SiNPs group when compared with the control. The genes expression of *IL6R*, *JAK1* and *TF,* were elevating in SiNPs group, miR-451a inhibitor + control group and miR-451a inhibitor + SiNPs group while were decreasing in miR-451a mimics + control group and miR-451a mimics + SiNPs group significantly when compared with the control. The genes expression of *STAT3*, *FGA* and *FGB* were elevating in SiNPs group, miR-451a inhibitor + control group and miR-451a inhibitor + SiNPs group. The gene expression of *FGG* was decreasing in SiNPs group, miR-451a mimics + control group and miR-451a mimics + SiNPs group. The results in vitro were almost consistant with that in vivo.

## Discussion

With an ever-increasing development of nanoparticles, the potential risks of these materials on human health and environmental safety is of great concern. As nanoparticles were widely used in nanomedicine, the nanoparticles could enter into target tissues via vascular endothelium [24]. We previously demonstrated that SiNPs could induce vascular endothelial damage [25]. However, the molecular mechanisms and subsequent toxicological outcomes of SiNPs-induced endothelial damage required further elucidation. In this study, we demonstrate the relationship between miR-451a and IL6R/STAT/TF signaling pathway in the process of SiNPs-induced endothelial dysfunction and pre-thrombotic state.

Repeated intratracheal instillation of SiNPs over 30 days in rats was accompanied by a reduction tendency regards to aortic blood flow velocity and cardiac volume (Fig. 2). Doppler traces showed the velocities present and temporal pattern of flow through the cardiac cycle with peak velocity. Blood flow controls the transport of zymogens, co-factors, enzymes and inhibitory factors to regulate coagulation and fibrin formation within a growing thrombus [26, 27]. In the presence of a growing thrombosis, the flow velocity is increasing in the narrow part of the vessel, and the total flow rate decreases because of the clot resistance. The rate of mass transfer in coagulation reactions determines the rate of thrombin generation, fibrin deposition and thrombi growth [28]. The interstitial space between platelets regulates the transport of coagulation proteins and platelet agonists into and out of thrombi, ultimately determining its architecture and arrest [29]. Thus, the hemodynamic changes induced by SiNPs would be expected to promote coagulation and reduce fibrinolytic function, ultimately increasing the propensity of the blood towards hypercoagulation.

Endothelial damage has been an important trigger for coagulation processes. Vascular endothelial cells showed severe damage with increasing concentration of SiNPs (Fig. 3). The oxidative stress and inflammatory response have been verified as key underlying mechanisms of nanomaterials toxicity. In this study, the ultrastructural and histopathological alteration to endothelial layers were accompanied by both oxidative stress and inflammation of the vessel issues in a dose-dependent manner (Fig. 4). The surface of SiNPs exhibit unsaturated bonds and different bonding states of hydroxyl which engenders a high capacity of particles to produce reactive oxygen species (ROS) and cause oxidative damage. ROS can activate nuclear transcription factors-κB (NF-κB) and c-Jun N-terminal kinase (JNK) signaling pathway, mediate inflammation, endothelial dysfunction and endothelial damage, contributing to the increased expression of proinflammatory markers and blood coagulation factors [30]. Our previous study also found that SiNPs could induce oxidative stress and reduce the activity of antioxidant enzymes, with increasing expression of interleukin-6 (IL-6) and tumor necrosis factor-α (TNF-α), both in vitro and in vivo [31]. Endothelial damage was also associated with imbalances in nitrite levels. Blood nitrite levels have been shown to inversely correlate with platelet aggregation, and positively correlate with bleeding time [32]. As well as deriving from arginine via eNOS, NO can also be generated by a two-step reduction pathway, in which nitrate is converted first into nitrite and then into NO. Thus, the NO/NOS system might promote the thrombotic pathways and blood clotting.

Additionally, the expression of related coagulation factors PECAM-1 and TF were activated by SiNPs (Fig. 4 and Additional file 1: Figure S1). PECAM-1 is an adhesion molecule which is activated as the result of inflammation. While TF is activated in response to vascular endothelial injury and/or hypercoagulation. Our results indicated that SiNPs could impact the coagulation function via activating the endogenous/exogenous coagulation system and fibrinolytic system (Fig. 5). Our previous study showed that endothelial cells could express TF and activate FVII response to vascular endothelial injury, which then combined to the form of TF-FVIIa complex at the site of endothelial cells [33]. Damaged endothelial cells could also release von willebrand factor (vWF) which promotes the platelet activation, adhesion and aggregation. SiNPs could also elevate the expression of fibrinogen and soluble E-selectin in plasma which indicated the coagulation dysfunction [34]. Thrombin is a fundamental mediator of coagulation cascade. Only 0.5% of blood coagulation factor (VII) is activated (FVIIa). This factor could rapidly activate Factor X (FXa) under the presence of phospholipids and Ca^2+^. FXa activates clotting factors to promote the blood coagulation cascade sequentially [35]. We suggested it is necessary to explore the underlying mechanism of SiNPs on coagulation fuction and thrombus formation.

The dual-luciferase reporter gene assay and microarray analysis were further conducted (Fig. 6). While miR-451 has two subunits (miR-451a and miR-451b), according to our results, only miR-451a had detected the target relationship with gene IL-6R. Thus, we extrapolated that miR-451a might be the subunit which has the biological effects. Based on our data, the miR-451a has a target regulatory relationship with *IL6R* and has a closely relationship with JAK/STAT/TF signaling pathway. After verified the results in vivo and in vitro (Fig. 7-9), miR-451a was confirmed as a regulator of IL6R in SiNPs-induced blood hypercoagulation. The expression of miR-451a in coronary sinus samples from patients with congestive heart failure were decreased significantly [36]. Downregulation of miR-451 promotes acute proliferation of macrophages and smooth muscle cells, thereby inducing tissue-engineered vascular graft (TEVG) stenosis and contribute to the development of hypertrophic cardiomyopathy (HCM) [37]. To the contrary, upregulation of endogenous miR-451 targeting endothelial cells (ECs) is involved in the modulation of myocardial ischemia-reperfusion (MI/R) injury [38]. So, the increasing miR-451 might be a biomarker of prognosis in clinical therapy of heart failure [39]. IL-6 can phosphorylate JAK and cause STAT3 up-regulation through glycoprotein 130 (GP130/IL6ST). IL-6 family play important roles in pathological process of cardiovascular diseases, and the IL6R-mediated JAK/STAT signaling pathway has been shown to participate in angiogenesis regulation and inflammation response [40].

In our study, miR-451a was detected as a possible key miRNA in the process of coagulation induced by SiNPs. Our previous study showed that the IL-6-mediated JAK/STAT signaling pathway played an important role in toxic effect of SiNPs-treated in zebrafish [41]. Here, the mimics/inhibitors of miR-451a transfected into SiNPs-treated endothelial cells was performed. The IL6R expression was reduced markedly by mimics of miR-451a, whereas miR-451a inhibitors obviously elevated the level of IL6R. Therefore, we could infer that miR-451a may interfere with the IL6R-STAT3 pathway to regulate the endothelial dysfunction and prethrombotic state induced by SiNPs. However, the protein corona-mediated alteration by biosurface interaction of SiNPs may suppress acute toxicity [42]. A cryopellet containing silica can activate intrinsic blood coagulation in rotational thromboelastometry [43]. When nanocarrier materials enter the body, it will contact with the blood inevitably and have various reactions with blood cells and proteins in blood system. It can be recognized by body immune system to generate complex immune responses. Also, high surface charge and low density of nanoparticles can induce platelet aggregation and activation, and result in thrombosis by activating endogenous coagulation pathway [44]. Therefore, our study privided one of molecular mechanism of hypercoagulation induced by nanoparticles which could offer a new thought to solve the blood toxicity of nanoparticles. The further research on the biosurface between nanoparticles and blood components is also required.

## Conclusions

For the first time, this study demonstrated that SiNPs could trigger the vascular endothelial dysfunction and a prethrombotic state via miR-451a negative regulating the IL6R/STAT/TF signaling pathway. Our data suggested that miR-451a could be a useful molecular target for countering the potential side-effects of promising nanoparticle-based biomedical applications.

## Additional file

Additional file 1: **Figure S1.** Effects of SiNPs on cellular adhesion molecule expression in SD rats’aortic arch sections were detected by immunohistochemical analysis. The expression of PECAM-1was increasing in a dose-dependent manner compared to the control group. (A) Control (B) 1.8 mg/kg·bw (C) 5.4 mg/kg·bw (D) 16.2 mg/kg·bw. **Table S1.** Primers used for qRT-PCR. **Table S2.** Hydrodynamic size and zeta potential of SiNPs in distilled water, DMEM, DMEM (10% serum) and normal saline as dispersion medium at different time points.**Table S3.** The top microRNA-gene ranked by degree over 5 in Signal-Net analysis. **Table S4** The summary of 16 significant pathways involved in 11 microRNA. (PDF 1631 kb)
