# Supplementary material for: Silica nanoparticles trigger the vascular endothelial dysfunction and prethrombotic state via miR-451 directly regulating the IL6R signaling pathway
Source: Part Fibre Toxicol. 2019 Apr 11;16:16. doi: 10.1186/s12989-019-0300-x (PMC6460825; doi:10.1186/s12989-019-0300-x)
Supplement: Supplementary file 1 — Figure S1. Effects of SiNPs on cellular adhesion molecule expression in SD rats’aortic arch sections were detected by immunohistochemical analysis. The expression of PECAM-1was increasing in a dose-dependent manner compared to the control group. (A) Control (B) 1.8 mg/kg·bw (C) 5.4 mg/kg·bw (D) 16.2 mg/kg·bw. Table S1. Primers used for qRT-PCR. Table S2. Hydrodynamic size and zeta potential of SiNPs in distilled water, DMEM, DMEM (10% serum) and normal saline as dispersion medium at different time points.Table S3. The top microRNA-gene ranked by degree over 5 in Signal-Net analysis. Table S4 The summary of 16 significant pathways involved in 11 microRNA. (PDF 1631 kb) [file 12989_2019_300_MOESM1_ESM.pdf]

Supplementary Information

Silica nanoparticles trigger the vascular endothelial dysfunction and prethrombotic state via miR-451 directly regulating the IL6R signaling pathway

Lin Feng[1,2], Xiaozhe Yang[1,2], Shuang Liang[1,2], Qing Xu[3], Mark R. Miller[4], Junchao Duan[1,2,*], Zhiwei Sun[1,2,*]

[1] Department of Toxicology and Sanitary Chemistry, School of Public Health, Capital Medical University, Beijing 100069, P.R. China

[2] Beijing Key Laboratory of Environmental Toxicology, Capital Medical University, Beijing 100069, P.R. China

[3] Core Facilities for Electrophysiology, Core Facility Center, Capital Medical University, Beijing 100069, P.R. China

[4] University/BHF Centre for Cardiovascular Science, Queens Medical Research Institute, The University of Edinburgh, Edinburgh, UK

Microarray and Bioinformatics analysis

For Affymetrix® microarray profiling, the total RNA was isolated from 30 zebrafish embryos per SiNPs-treatment group (3 ng/nL) by the TRIzol reagent (Invitrogen, Carlsbad, Canada) and purified with an RNeasy Mini Kit (Qiagen, Hilden, Germany) according to the manufacturer's protocol. The amount and quality of RNA were determined by a UV-Vis Spectrophotometer (Thermo, NanoDrop 2000, USA) at the absorbance of 260 nm. The mRNA expression profiling was measured using Zebrafish Gene 1.0 ST Array (Affymetrix GeneChip®, USA), which contains 59,302 gene-level probe sets. The microarray analysis was performed by Affymetrix® Expression Console Software (version 1.2.1). Raw data (CEL files) were normalized at transcript level using robust multi-array average method (RMA workflow). Median summarization of transcript expressions was calculated. Gene-level data was then filtered to include only those probe sets that are in the 'core' meta probe list, which represent RefSeq genes.

For microarray data analysis, differential expression genes were identified based on random variance model (RVM) t-test. And the differential expression genes were considered to be up or down regulated with at least $p < 0.05$. Genes with similar expression patterns often facilitate the overlapping functions. Accordingly, the cluster analysis of gene expression patterns was analyzed by Cluster and Java Treeview software. Pathway analysis was used to find out the significant pathway of the differential genes according to Kyoto Encyclopedia of Genes and Genomes (KEGG) database, Biocarta and Reatome databases. Fisher's exact test was performed to select the significant pathway, and the threshold of significance was considered as $p < 0.05$ (Duan et al., 2016).

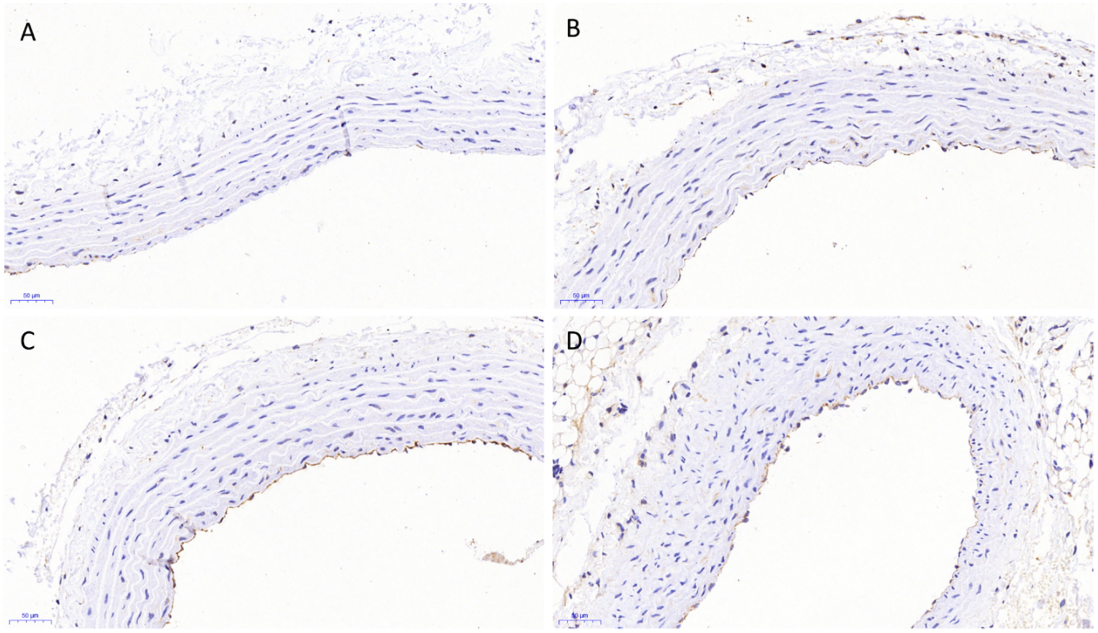

Supplementary Figure 1. Effect of SiNPs on cellular adhesion molecule expression in SD rats' aortic arch sections were detected by immunohistochemical analysis. The expression of PECAM-1 was increasing in a dose-dependent manner compared to the control group. (A) Control (B) 1.8 mg/kg·bw (C) 5.4 mg/kg·bw (D) 16.2 mg/kg·bw

Table S1 Primers used for qRT-PCR

Name	Species	Forward	Reverse
miR-451a	human	cgcgAAACCGTTACCATTACTGAGTT	--
Jak1	rat	TGGAACCAACGACAATGAGC	CTCCAGTGAGCTGGCATCAA
Stat3	rat	CGGAGAAGCATCGTGAGTGA	TCCAATGCAGGCAATCTGTT
Tf	rat	TCAGCAGAGACCACCGAAGA	CAAGACAGGCACCAGACCAC
Il6r	rat	TCCAGCAACGAGGAGAATGA	GGTGGACTCGGACCTTGAGA
Fib	rat	AAGGAGGACGCTCTGGTCAC	GATTCCACACGCGGTACTCA
Vwf	rat	TGCCGCCTGCCACAACAATATC	CCTGGAAGATGTCACTGGTAAGGATTC
β-actin	rat	GATGCGGAAACTGGCAAAGG	GAGGGCAAAGTGGTAAACGC
JAK1	human	TGGAACCAACGACAATGAGC	CTCCAGTGAGCTGGCATCAA
STAT3	human	CGGAGAAGCATCGTGAGTGA	TCCAATGCAGGCAATCTGTT
TF	human	ACTATGAGTTGCTGTGCCTTGATGG	GTGCTGCTGTTGACGTAATATCTTGTG
IL6R	human	GCTGTGCTCTTGGTGAGGAAGTTT	CTGAGCTCAAACCGTAGTCTGTAGAAA
FGA	human	GGCAATGACTACCTCCACTTACTAACC	ATAGCCTTCAGCCTCAGAGCCTAC
FGB	human	ACCTTCGTGTGCTTCGTTCAATCC	TGCCAGACACCACAGGAATATTGC
FGG	human	GAGTGGAACTGGAAGACTGGAATGG	GCGGTACTTGTCAGCTTCAGGTC
β-ACTIN	human	GATGCGGAAACTGGCAAAGG	GAGGGCAAAGTGGTAAACGC

Table S2 Hydrodynamic size and zeta potential of SiNPs in distilled water, DMEM, DMEM (10% serum) and normal saline as dispersion medium at different time points.

Time (h)	Hydrodynamic Size (nm)				Zeta (mV)			
	Distilled Water	DMEM	DMEM (10 % serum)	Normal Saline	Distilled Water	DMEM	DMEM (10 % serum)	Normal Saline
0	117.90±1.15	119.37±0.45	306.93±8.80	116.67±1.27	-32.70±1.65	-24.00±0.62	-8.07±0.86	-30.77±1.15
3	117.87±0.42	119.17±0.71	201.27±6.10	118.40±0.92	-32.87±3.32	-25.93±1.12	-10.13±0.12	-34.33±1.86
6	118.13±1.68	119.73±0.76	197.87±1.36	118.40±0.56	-33.83±2.45	-26.03±0.51	-10.06±0.16	-32.93±0.58
12	118.97±0.31	135.07±3.86	131.77±1.50	116.13±0.76	-32.33±3.37	-24.27±1.68	-23.47±0.80	-28.60±1.68
24	118.27±0.58	139.77±1.80	133.27±1.19	115.97±1.10	-31.57±2.63	-22.60±0.92	-22.57±1.50	-21.80±1.65

Table S3 The top microRNA-gene ranked by degree over 5 in Signal-Net analysis.

microRNA	Regulation	Target	Degree
miR-146a	up	zgc:113337, nrxn2a, zgc:158291, zgc:165525, zgc:172014, si:ch211-236k19.4, rdh5, glra4b, zgc:175145, thop1, ghrb, entpd3, zswim6, crygs1, rlbp1b, atp2a1l, zwi, zgc:162161, zgc:113390, nrld4a, opn1sw2, dio2, tdrd6, prph2b, opn1lw2, slc7a2, pdca, pdpk1a, apln, slc23a1, trim9, wnt11r, slc47a1, impdh1a	34
miR-31	up	pcdh2aa3, pcdh2ab2, zgc:153102, zgc:73075, pvalb9, zgc:113307, zgc:103625, grk1b, anxa2b, cyp1c1, dpys, igf2a, zwi, zgc:86725, LOC570112, si:ch211-163l21.8, LOC565734, rev3l, plcd3b, LOC563546, rx1, cygb1, lum, aspa, dcun1d4, pdpk1a, trh, apln, adss, slc25a12	30
miR-146b	up	zgc:113337, zgc:165525, zgc:172014, si:ch211-236k19.4, glra4b, zgc:175145, thop1, ghrb, entpd3, zswim6, crygs1, rlbp1b, atp2a1l, si:ch211-216l23.2, zwi, zgc:162161, zgc:113390, opn1sw2, dio2, tdrd6, prph2b, opn1lw2, pdca, pdpk1a, apln, impdh1a	26
miR-29a	up	dpysl5a, si:dkey-61p9.6, zgc:114120, cyp1c1, kmo, igf2a, cahz, zswim6, si:ch211-216l23.2, rev3l, si:ch73-142c19.2, LOC100007655, igfbp2a, csrp1b, prph2a, dcun1d4, si:dkey-108d22.4, lcn15, ppp2r2c, sh3gl2, c1qtnf1, dpysl5b, trim9, clc47a1	24
miR-21	up	zgc:77058, zgc:73075, klf2a, cyp1c1, scn8aa, igsf21b, nr1d4a, LOC100332446, tmem229b, gck, cygb1, rpa1, dio2, wscd2, hmgcs1, si:dkey-246g23.4, camsap1a, zgc:158645, spon1a, pde6c, guca1a, clrn1, agtr2	23
miR-222b	up	atpla3b, entpd2a.1, grin1b, reep6, LOC569661, ca4b, wu:fb99g09, plcd3b, tmem229b, cygb1, mao, iqcb1, elovl4b, dgat1a, wscd2, si:dkey-246g23.4, trim13, slc23a1, fstl4, pde6a, gnat2	21
miR-729	down	zgc:103536, capg, homez, zgc:56382, gsnb, lgmn, gga3, pld2, we:fe11b02, foxj1a, dusp1, ctsc, rnaset2, hars2, zgc:85866, hdlbp, ephx2, mtmr7a, tpm3	19
miR-451	down	il6r, slc6a13, zgc:56197, trap1, eif6, zgc:110699, LOC100004826, LOC100330405, LOC568379, mxc, stat3, snapin, camk4, acsl4l, fos, txndc5	16
miR-726	down	zgc:112964, zgc:92360, zgc:174888, si:ch211-239j19.3, ptges, igfbp1a, hoxb5b, musk, stk351, ahnak, mxc, gnpnat1, irf9, asap3, chd1l	15
miR-7147	down	nfkbiaa, id3, zgc:114164, ctsba, foxj1a, slc25a33, hig1, cebpd	8
miR-193a	down	zgc:91909, LOC100007087, hbegfa, zgc:136338	5

Table S4 The summary of 16 significant pathways involved in 11 microRNA.

Pathway name	microRNA	Degree
Metabolic pathways	miR-222b, miR-21, miR-146b, miR-146a	6
Purine metabolism	miR-146b, miR-146a, miR-222b, miR-31	5
Calcium signaling pathway	miR-146a, miR-222b, miR-31	4
Tryptophan metabolism	miR-21, miR-29a, miR-222b	4
Herpes simplex infection	miR-7147, miR-451, miR-726	3
Phototransduction	miR-21, miR-31, miR-222b	3
Adipocytokine signaling pathway	miR-451, miR-7147	2
Salmonella infection	miR-451, miR-193a	2
Toll-like receptor signaling pathway	miR-7147, miR-451	2
Arachidonic acid metabolism	miR-726, miR-729	2
JAK-STAT signaling pathway	miR-451, miR-726	2
MAPK signaling pathway	miR-729, miR-451	2
Neuroactive ligand-receptor interaction	miR-21, miR-222b	2
Apoptosis	miR-7147	1
Cardiac muscle contraction	miR-222b	1
Glycine, serine and threonine metabolism	miR-222b	1

Reference

Junchao Duan, Yang Yu, Yang Li, Hongcui Liu, Li Jing, Man Yang, Ji Wang, Chunqi Li, Zhiwei Sun. Low-dose exposure of silica nanoparticles induces cardiac dysfunction via neutrophil-mediated inflammation and cardiac contraction in zebrafish embryos. *Nanotoxicology*. 2016;10(5):575-585.
